# WSD-0922, a novel brain-penetrant inhibitor of epidermal growth factor receptor, promotes survival in glioblastoma mouse models

**DOI:** 10.1093/noajnl/vdad066

**Published:** 2023-05-27

**Authors:** Jason E Conage-Pough, Sylwia A Stopka, Ju-Hee Oh, Ann C Mladek, Danielle M Burgenske, Michael S Regan, Gerard Baquer, Paul A Decker, Brett L Carlson, Katrina K Bakken, Jinqiang Zhang, Lily Liu, Claire Sun, Zhihua Mu, Wei Zhong, Nhan L Tran, William F Elmquist, Nathalie Y R Agar, Jann N Sarkaria, Forest M White

**Affiliations:** Department of Biological Engineering, Massachusetts Institute of Technology, Cambridge, Massachusetts, USA; David H. Koch Institute for Integrative Cancer Research, Massachusetts Institute of Technology, Cambridge, Massachusetts, USA; Center for Precision Cancer Medicine, Massachusetts Institute of Technology, Cambridge, Massachusetts, USA; Department of Neurosurgery, Brigham and Women’s Hospital, Harvard Medical School, Boston, Massachusetts, USA; Department of Radiology, Brigham and Women’s Hospital, Harvard Medical School, Boston, Massachusetts, USA; Department of Pharmaceutics, University of Minnesota, Minneapolis, Minnesota, USA; Department of Radiation Oncology, Mayo Clinic, Rochester, Minnesota, USA; Department of Radiation Oncology, Mayo Clinic, Rochester, Minnesota, USA; Department of Neurosurgery, Brigham and Women’s Hospital, Harvard Medical School, Boston, Massachusetts, USA; Department of Neurosurgery, Brigham and Women’s Hospital, Harvard Medical School, Boston, Massachusetts, USA; Department of Biomedical Statistics and Informatics, Mayo Clinic, Rochester, Minnesota, USA; Department of Radiation Oncology, Mayo Clinic, Rochester, Minnesota, USA; Department of Radiation Oncology, Mayo Clinic, Rochester, Minnesota, USA; Wayshine Biopharm, Corona, California, USA; Wayshine Biopharm, Corona, California, USA; Wayshine Biopharm, Corona, California, USA; Wayshine Biopharm, Corona, California, USA; Wayshine Biopharm, Corona, California, USA; Department of Cancer Biology, Mayo Clinic, Scottsdale, Arizona, USA; Department of Pharmaceutics, University of Minnesota, Minneapolis, Minnesota, USA; Department of Neurosurgery, Brigham and Women’s Hospital, Harvard Medical School, Boston, Massachusetts, USA; Department of Radiology, Brigham and Women’s Hospital, Harvard Medical School, Boston, Massachusetts, USA; Department of Cancer Biology, Dana-Farber Cancer Institute, Boston, Massachusetts ¸ USA; Department of Radiation Oncology, Mayo Clinic, Rochester, Minnesota, USA; Department of Biological Engineering, Massachusetts Institute of Technology, Cambridge, Massachusetts, USA; David H. Koch Institute for Integrative Cancer Research, Massachusetts Institute of Technology, Cambridge, Massachusetts, USA; Center for Precision Cancer Medicine, Massachusetts Institute of Technology, Cambridge, Massachusetts, USA

**Keywords:** blood-brain barrier, EGFR, erlotinib, glioblastoma, WSD-0922

## Abstract

**Background:**

Although the epidermal growth factor receptor (EGFR) is a frequent oncogenic driver in glioblastoma (GBM), efforts to therapeutically target this protein have been largely unsuccessful. The present preclinical study evaluated the novel EGFR inhibitor WSD-0922.

**Methods:**

We employed flank and orthotopic patient-derived xenograft models to characterize WSD-0922 and compare its efficacy to erlotinib, a potent EGFR inhibitor that failed to provide benefit for GBM patients. We performed long-term survival studies and collected short-term tumor, plasma, and whole-brain samples from mice treated with each drug. We utilized mass spectrometry to measure drug concentrations and spatial distribution and to assess the impact of each drug on receptor activity and cellular signaling networks.

**Results:**

WSD-0922 inhibited EGFR signaling as effectively as erlotinib in in vitro and in vivo models. While WSD-0922 was more CNS penetrant than erlotinib in terms of total concentration, comparable concentrations of both drugs were measured at the tumor site in orthotopic models, and the concentration of free WSD-0922 in the brain was significantly less than the concentration of free erlotinib. WSD-0922 treatment provided a clear survival advantage compared to erlotinib in the GBM39 model, with marked suppression of tumor growth and most mice surviving until the end of the study. WSD-0922 treatment preferentially inhibited phosphorylation of several proteins, including those associated with EGFR inhibitor resistance and cell metabolism.

**Conclusions:**

WSD-0922 is a highly potent inhibitor of EGFR in GBM, and warrants further evaluation in clinical studies.

**Key Points**

1. WSD-0922 demonstrates promise for GBM in preclinical studies.
2. Although BBB-penetrant, WSD-0922 has limited free-drug availability within the brain.
3. Additional target specificity of WSD-0922 may enhance anti-EGFR efficacy.

**Importance of the Study**

There has been minimal improvement in clinical outcomes for GBM patients, even though significant progress has been made in the genetic characterization of the disease. EGFR is a relevant biomarker for nearly half of all GBMs; however, efforts to target this protein have had limited impact on patient survival. This lack of efficacy is likely due to several factors, including the poor CNS penetrance of most EGFR-targeting agents. In the present study, we demonstrated that the novel EGFR inhibitor WSD-0922 is highly effective at inhibiting the EGFR signaling pathway in in vitro and in vivo settings. Additionally, WSD-0922 provided a profound increase in survival for the GBM39 orthotopic PDX model, and suppressed phosphorylation of proteins associated with EGFR inhibitor resistance. Our data suggest WSD-0922 is a promising candidate for further clinical studies and may provide therapeutic benefits for a subset of GBM patients.

Despite being a well-characterized genetic marker of glioblastoma (GBM, CNS WHO grade 4),^1^ the epidermal growth factor receptor (EGFR) remains an elusive therapeutic target in the disease. EGFR is amplified or mutated in nearly 50% of GBMs,^2^ and is the defining signature of the classical subtype.^3^ Even so, a broad spectrum of drugs targeting EGFR, ranging from small molecules to antibody-based therapies, has failed to significantly prolong GBM patient survival.^4^ This lack of efficacy has been attributed to a myriad of factors including the poor blood-brain barrier (BBB) penetrance of most inhibitors, involvement of efflux transporters, adaptive signaling responses and drug resistance, disease heterogeneity, and the presence of EGFR mutations in GBM that are distinct from those seen in diseases more amenable to EGFR inhibition.^5–7^ These obstacles have led many to dismiss EGFR-targeted therapy as a viable strategy for treating GBM.

The small molecule kinase inhibitor WSD-0922 was recently developed to address many of these challenges. Most small molecule inhibitors of EGFR are ATP-mimetics that competitively block the ATP-binding pocket of the receptor. This mode of inhibition frequently leads to the emergence of resistance through mutations in the described region.^8^ To counter this, WSD-0922 was designed as a noncompetitive inhibitor of the ATP-binding domain, with good brain penetrance and poor substrate specificity for common efflux transporters.^9^ These characteristics make WSD-0922 a promising candidate for further preclinical evaluation.

The goal of this study was to comprehensively characterize WSD-0922, assessing in vitro and in vivo efficacy, along with pharmacokinetics and pharmacodynamics (PK/PD), and drug distribution. We compared WSD-0922 to erlotinib, a potent, well-characterized first-generation ATP-competitive inhibitor of EGFR.^10,11^ Although erlotinib received clinical approval for treatment of non-small cell lung cancer and pancreatic cancer, clinical trials for GBM have not been successful,^12,13^ potentially due to poor penetration across the BBB. We demonstrate that WSD-0922 effectively inhibits EGFR signaling across multiple GBM patient-derived xenograft models (PDX). Strikingly, mice from one of the intracranial PDX models had a substantial increase in survival with WSD-0922 treatment. Upon further investigation, this model had a unique signaling axis of metabolic enzymes and cofilin whose phosphorylation was diminished with WSD-0922. Our data strongly support the ongoing clinical evaluation of WSD-0922 and the need to identify a potential subset of GBM patients who may benefit from this anti-EGFR therapy.

## Materials and Methods

### Animal Studies

GBM PDX lines were established, maintained, and used in experiments as previously described.^14,15^ Studies were approved by the Mayo Clinic Institutional Animal Care and Use Committee, and all animal care procedures were followed. For phosphoproteomics, MALDI-MSI, and concentration measurements in plasma, female athymic nude mice (Charles River, strain code 553) were sacrificed after 3 doses of erlotinib or 5 doses of WSD-0922. For long-term survival studies, short-term explant cultures were grown in serum-free conditions (see Supplementary Materials) and mice were injected intracranially with 300 000 (GBM6, GBM39) or 100 000 (GBM12) cells, randomized (10 mice per group), and treated with erlotinib or WSD-0922 7–14 days later. Drugs were administered by oral gavage, and mice were observed daily by staff blinded to the treatment groups until reaching a moribund state. Erlotinib was dosed daily Monday–Sunday at 80 mg/kg, while WSD-0922 was dosed twice daily at 40 mg/kg Monday–Sunday (GBM12, GBM39) or twice daily at 40 mg/kg Monday–Friday and once daily Saturday–Sunday (GBM6). Survival was defined as the time from tumor implantation to a moribund state in the mice. Differences in survival across groups were assessed by the Log-Rank test.

### Liquid Chromatography-Tandem Mass Spectrometry

The analysis of erlotinib drug concentrations was performed as previously described with slight modification.^16^ Erlotinib and WSD-0922 were extracted from the mouse plasma and brain homogenate using a liquid–liquid extraction method. Briefly, brain specimens were homogenized in 2 volumes of ice-cold 5% bovine serum albumin. 50 μL aliquots of the plasma and brain homogenate were spiked with 5 ng of the internal standard (ie, deuterium-labeled erlotinib (erlotinib-d6) for erlotinib and gefitinib for WSD-0922, respectively). Then extraction of erlotinib was carried out by adding 2 volumes of pH 11 buffer solution and ten volumes of ethyl acetate, whereas 1 N sodium hydroxide and ethyl acetate were added for the extraction of WSD-0922. The as-prepared samples were vigorously vortexed for 5 minutes and centrifuged at 14 000 rpm for 5 minutes at 4 °C. After freezing the aqueous phase at −80 °C for 20 minutes, the organic phase was decanted into a 1.5 mL microcentrifuge tube and dried under nitrogen gas. The dried residue was reconstituted in 100 µL of the mobile phase. Chromatographic analysis was performed with an Agilent 1200 series HPLC system (Agilent Technologies, Santa Clara, CA) equipped with a Synergi™ Polar-RP column (75 × 2 mm, 4 µm; Phenomenex, Torrance, CA). The mobile phase for the isocratic erlotinib analysis consisted of 0.1% formic acid in distilled water (A) and 0.1% formic acid in acetonitrile (B) (50:50, v/v), and it was continuously fed at a flow rate of 0.25 mL/min. For the analysis of WSD-0922, gradient elution at a flow rate of 0.5 mL/min was employed as follows: 0.00–0.50 minutes 32% B (isocratic), 0.50–0.75 minutes 32%–70% B (linear gradient), 0.75–1.75 minutes 70% B (isocratic), 1.75–2.00 minutes 70%–32% B (linear gradient), and 2.00–6.00 minutes 32% B (isocratic). The column effluent was monitored by a TSQ Quantum Classic mass spectrometer (Thermo Finnigan, San Jose, CA) with an electrospray interface in positive ion mode. The mass-to-charge ratio (*m/z*) transitions were 394.4 > 278.0, 400.1 > 278.0, 443.2 > 134.1, and 447.1 > 128.1 for erlotinib, erlotinib-d6, WSD-0922, and gefitinib, respectively.

### Sample Preparation for MALDI MSI

Flank tumors and whole brains were removed and flash-frozen in liquid nitrogen from GBM6, 12, and 39 mice dosed with either erlotinib, WSD-0922, or the corresponding vehicle. Using a cryo microtome the tissues were sectioned at 10 µm thickness and thaw mounted on indium-tin-oxide slides. Serial sections were collected onto regular microscope slides for hematoxylin and eosin (H&E) staining. The H and E stained tissue sections were digitally imaged with bright field microscopy using a 20 × magnification plan-apochromat lens (Zeiss Observer Z.1, Oberkochen, Germany) and a stitching algorithm to create a high-resolution image of each tissue section.

### Drug Quantification Using MALDI MSI

Erlotinib and WSD-0922 standards were dissolved in DMSO and spiked into mouse brain tissue homogenates at varied concentrations. Six concentrations, including a blank, were selected for erlotinib (1.53 µM to 145.23 µM) and WSD-0922 (1.36 µM to 151.08 µM). The homogenate mixtures were vortexed and dispensed into a gelatin tissue microarray (TMA) mold and frozen. The TMA block was then sectioned at 10 µm thickness and thaw-mounted onto the same indium-tin-oxide slide as the tissue sections. A matrix solution composed of 2,5-dihydroxybenzoic acid (160 mg/mL) was dissolved in 70:30 methanol: 0.1% TFA with 1 % DMSO and sprayed using a TM-sprayer (HTX imaging, Carrboro, NC). Spraying parameters consisted of a two-pass cycle with a flow rate of (0.18 mL/min), spray nozzle velocity (1200 mm/min), spray nozzle temperature (75 °C), nitrogen gas pressure (10 psi), track spacing (2 mm). Recrystallization was then performed with 5% acetic acid at 85 °C for 6 minutes.

A timsTOF fleX mass spectrometer (Bruker Daltonics, Billerica, MA) operating in positive ion mode scanning a range of *m/z* 100-2000 was used to monitor [erlotinib+H]^+^ and [WSD-0922+H]^+^ ion distribution in both the flank and brain samples. Detection of erlotinib and WSD-0922 was optimized by infusing drug standards through ESI to adjust transfer funnels, quadrupole, collision cell, and focus pre-TOF parameters. The ESI source was also used to mass calibrate each drug method using the Agilent tune mix solution (Agilent Technologies, Santa Clara, CA). Both ESI methods were then applied to the MALDI source parameters, including a step size of 100 µm, a laser repetition of 10 000 Hz, and 1000 laser shots per pixel. Data visualization were performed using SCiLS Lab software (version 2021a premium, Bruker Daltonics, Billerica, MA) with TIC normalization.

### Pixel-Wise Calibration of Drug Images

The ion images were exported to imzML using SCiLS Lab software and the R package rMSIproc (https://github.com/prafols/rMSIproc) (doi: 10.1093/bioinformatics/btaa142.) was used for data loading and visualization. An in-house R package was used for pixel-wise calibration of the erlotinib and WSD-0922 *m/z* intensity values to produce images with absolute concentration values at each pixel for comparison of quantitative distribution of the 2 drugs. The mean absolute intensity and standard deviation of each calibration spot (1.5 mm TMA spot) were computed and linear regression model (absolute intensity vs drug concentration) was fitted for each experimental run and then used for pixel-wise calibration.

### Phosphotyrosine Analysis of Tumor Samples

Tumors were homogenized by sonication in 8 M urea and protein concentration was measured by a bicinchoninic acid (BCA) assay (Pierce). Urea lysates were reduced with 10 mM dithiothreitol (DTT), alkylated with 55 mM iodoacetamide, and digested with trypsin overnight. Digested peptides were desalted using C18 cartridges (Waters) and were labeled with TMT 10plex or 11plex isobaric mass tags. TMT-labeled peptide samples were subjected to a previously described 2-step enrichment process,^17^ and the resulting phosphopeptides were loaded directly onto an in-house packed analytical capillary column (50 μm ID × 12 cm, 5 μm C18) with an integrated electrospray tip (1–2 μm orifice). Eluates were then subjected to LC-MS/MS as previously described on the Q-Exactive HF-X^17^ and the Orbitrap Exploris 480 with minor modifications. For Exploris analyses, full MS1 scans were acquired in the *m/z* range of 380–2000, with maximum injection time determined automatically and data-dependent acquisition performed with a 3-second cycle time. Limited LC-MS/MS analysis of the most abundant peptides to adjust for channel-to-channel loading variation was carried out on an Orbitrap Q-Exactive Plus mass spectrometer using ~15 ng of peptide. Supernatant was loaded onto an acidified trapping column and analyzed with gradients as follows: 0%–13% solvent B in 4 minutes, 13%–42% in 46 minutes, 42%–60% in 7 minutes, 60%–100% in 3 minutes, and 100% for 8 minutes, before equilibrating back to Solvent A. Full scans (MS1) were acquired in the *m/z* range of 350–2000 at a resolution of 70 000 (*m/z* 100). The top 10 most intense precursor ions were selected and isolated with an isolation width of 0.4 *m/z*. Selected ions were HCD fragmented at normalized collision energy of 33% at a resolution of 70 000.

Additional methods are described in Supplementary Materials.

## Results

### WSD-0922 is an ATP Non-competitive Inhibitor of Mutant and WT EGFR

**Figure 1.**
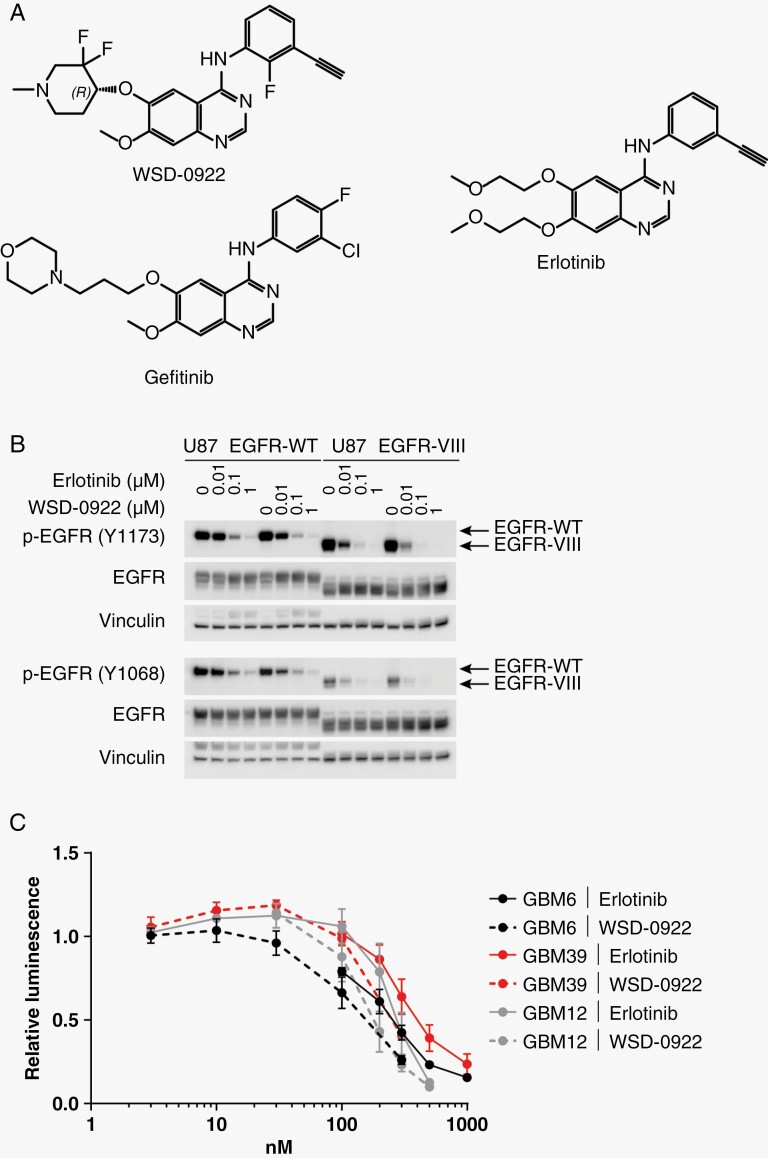
Biochemical and in vitro characterization of WSD-0922. (A) The chemical structure of WSD-0922 compared to the first-generation epidermal growth factor receptor (EGFR) inhibitors erlotinib and gefitinib. Structures were generated using ChemDraw 21.0.0. (B) Western blot showing the dose-dependent effect of erlotinib and WSD-0922 on pEGFR in U87 cell lines stably overexpressing WT or vIII EGFR. Antibodies against Tyr 1173 and Tyr 1068 pEGFR were used for immunoblotting. (C) Cell TiterGlo was used to assess the impact of erlotinib and WSD-0922 on the viability of GBM patient derived xenograft lines in ex vivo culture. Lines were treated with the indicated doses of drug for 14 days before luminescence readings were measured. Some data points were omitted to allow for curve fitting and determination of IC_50_ values.

WSD-0922 is a promising candidate for treating EGFR-driven tumors, including GBM. WSD-0922 was designed to bind non-competitively to EGFR, in a region distinct from ATP, thus avoiding mutations in the ATP-binding domain of EGFR that are a frequent source of eventual drug resistance.^18^ The structure of WSD-0922 is similar to other EGFR inhibitors (**Figure 1A**),^19,20^ however in vitro kinetic data confirm that WSD-0922 is likely a non-competitive inhibitor of EGFR (**Supplementary Figure 1B**). WSD-0922 demonstrated strong specificity for WT EGFR in in vitro kinase inhibition studies (**Table 1**), with a cell-free IC_50_ of 0.056 nM (**Supplementary Table 1**). These data compare favorably with erlotinib, which has previously demonstrated a low nanomolar IC_50_ in vitro.^21^ In addition to EGFR inhibition, WSD-0922 has specificity for Ephrin receptors, HER4, and Src Family Kinase (SFK) members (**Table 1**), targets that have been associated with EGFR tyrosine kinase inhibitor resistance^22,23^ and thus could augment the clinical effectiveness of the drug in GBM.

**Table 1.** WSD-0922 Inhibition of Epidermal Growth Factor Receptor and Other Kinases

Kinase	%Inhibition (1 µM WSD-0922)
EGFR	93.75
EPHA1	55.36
SRC	39.55
HCK	49.69
LCK	46.2
LYNa	70.94
HER4	69.77

We compared the efficacy of WSD-0922 to erlotinib in cell lines and ex vivo cultures. In U87 MG cells stably expressing WT and vIII forms of EGFR, WSD-0922 inhibited EGFR phosphorylation (pEGFR) to the same degree as erlotinib, as assessed by western blot (**Figure 1B**). For ex vivo cultures, we selected three primary patient-derived GBM lines, including GBM6 and GBM39, which predominantly express EGFRvIII, and GBM12, which contains a point mutation (G719A) in the EGFR tyrosine kinase domain. WSD-0922 and erlotinib had a similar effect on the viability of GBM PDX lines, with an EC50 of approximately 200 nM for GBM6, GBM39, and GBM12 (**Figure 1C**). Subsequent experiments with the GBM8 and GBM108 (WT EGFR amplified) PDX lines yielded an EC50 of 1 µM for both drugs (**Supplementary Figure 1C**).

### Assessment of WSD-0922 Pharmacodynamics and in vivo Inhibition of EGFR Signaling

**Figure 2.**
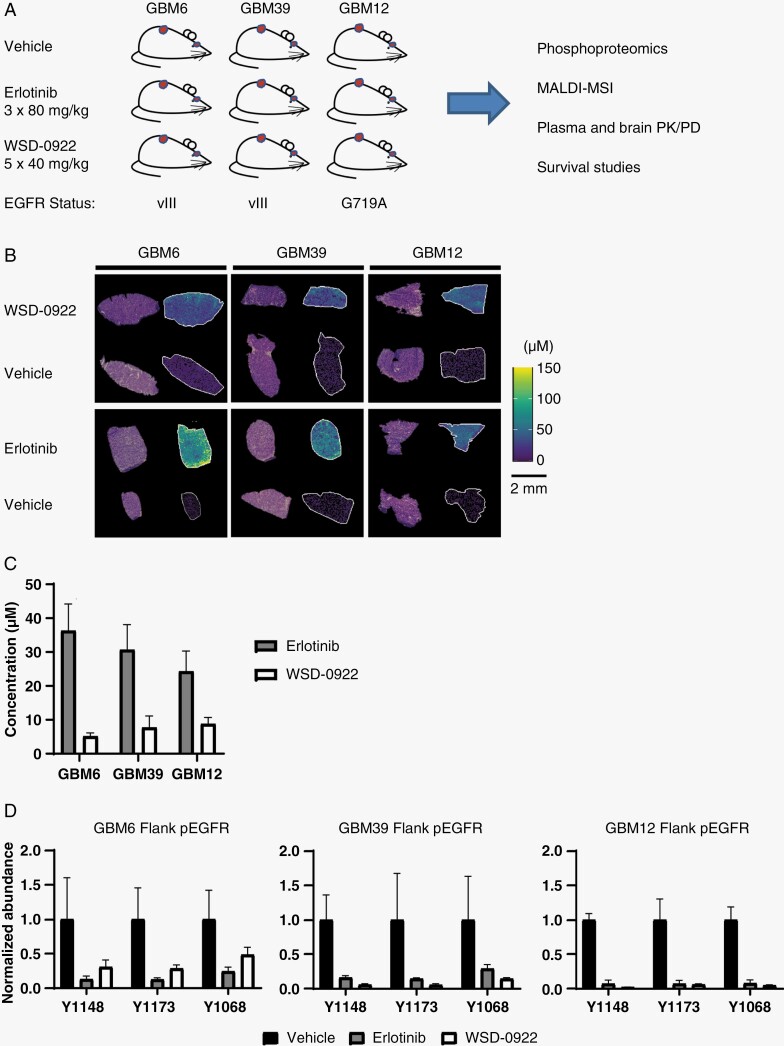
Flank tumor models demonstrate in vivo efficacy of WSD-0922. (A) Overview of the preclinical study and the modalities used to evaluate WSD-0922. (B) Representative MALDI-MS images showing distribution of erlotinib and WSD-0922 within flank tumors, after 3 × 80 mg/kg (erlotinib) or 5 × 40 mg/kg (WSD-0922) doses. MALDI-MS images are accompanied by corresponding H&E staining. (C) The concentration of drug in the plasma of each mouse was measured using liquid chromatography mass spectrometry. (D) Phosphoproteomics was used to measure the inhibition of epidermal growth factor receptor (EGFR) signaling within the flank tumors. Bars represent the relative abundance of phosphopeptides containing the indicated EGFR tyrosine phosphorylation sites upon drug treatment. Values are normalized to the average abundance for the vehicle treated tumors.

Given the comparable in vitro performance of WSD-0922 to erlotinib, we next sought to assess the in vivo efficacy of these two EGFR TKIs using GBM12, GBM39, and GBM6 PDX orthotopic and flank tumor models. To characterize the therapeutic response to these drugs, we collected plasma and tumor samples from each animal for pharmacokinetic/pharmacodynamic analysis (PK/PD), MALDI mass spectrometry imaging (MALDI-MSI), and phosphoproteomics analyses (**Figure 2A**).

We initially used the flank tumor models to evaluate drug efficacy in the absence of the BBB, predicting that drug distribution for WSD-0922 and erlotinib should be similar in these mice. After GBM6, GBM39, or GBM12 subcutaneous tumors were established, mice were treated with either erlotinib (80 mg/kg, three doses, QD) or WSD-0922 (40 mg/kg, five doses, BID). Mice were sacrificed two hours after the last dose of drug and tumors were resected for analysis. MALDI-MSI was used, along with external standards, to capture drug distribution in each tumor to provide spatially localized quantitative concentrations for each drug. Significantly lower concentrations of WSD-0922 were observed within GBM6 and GBM39 flank tumors when compared to erlotinib (**Figure 2B**, **Supplementary Figure 2** and **Table 2**). Consistent with the lower concentration of drug at the tumor site, WSD-0922 plasma concentrations were lower than erlotinib in these mice (**Figure 2C**, **Supplementary Table 3**). However, the difference in drug concentration between WSD-0922 and erlotinib in GBM6 and GBM39 did not affect the efficacy of the drug as determined by phosphoproteomics, with WSD-0922 inhibiting pEGFR as effectively as erlotinib (**Figure 2D**, **Supplementary Figure 3**). WSD-0922 and erlotinib also substantially downregulated phosphorylation of canonical downstream EGFR signaling targets, including GAB1, SHC1, and ERK1/2 (**Supplementary Figure 4**). These results suggest that regardless of differences in drug concentration within the tumor, both drugs are above a threshold concentration required for effective inhibition of EGFR kinase activity. Phosphoproteomic data further supported the affinity of WSD-0922 for additional kinases, with WSD-0922 preventing the phosphorylation of several targets that were upregulated in erlotinib-treated tumors (**Supplementary Figure 5 and Table 4**), including IGF-1R, SHB, and several SFK substrates. These data demonstrate the effectiveness of WSD-0922 against EGFR signaling in GBM patient-derived models and led us to evaluate whether WSD-0922 could provide a survival advantage in mice.

### WSD-0922 Promotes Survival in the GBM39 Intracranial Mouse Model

**Figure 3.**
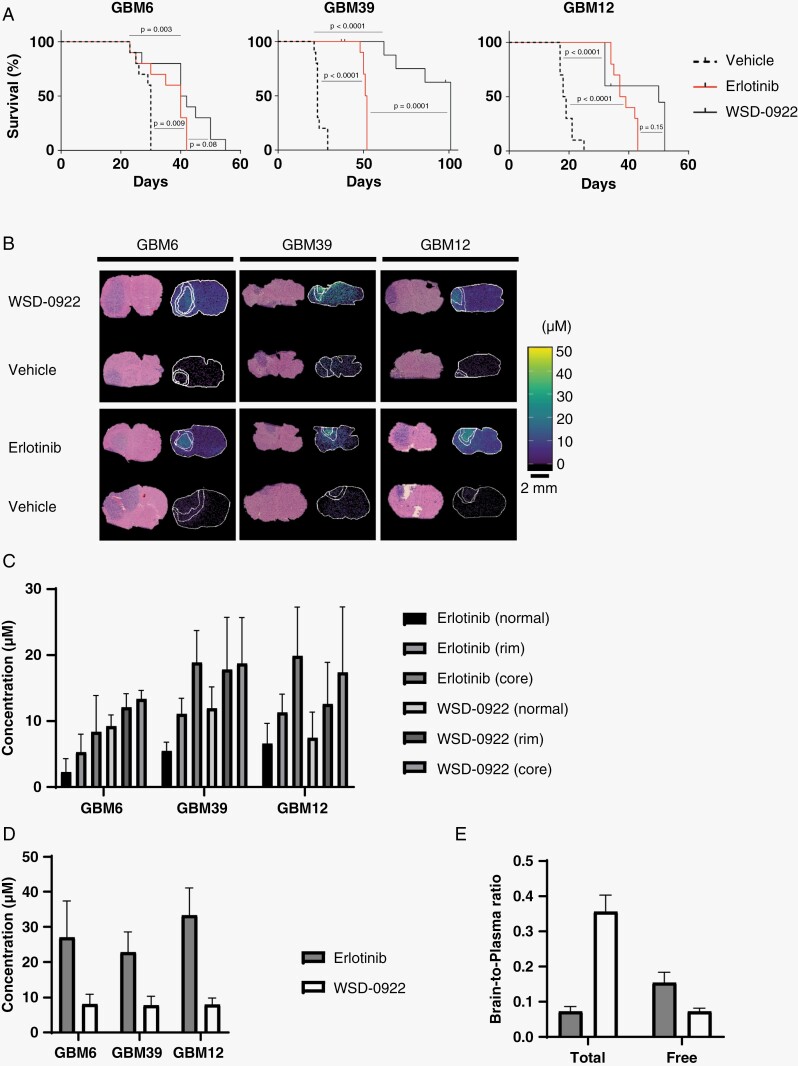
WSD-0922 prolongs survival in the GBM39 intracranial mouse model. (A) Kaplan–Meier curves showing the long-term survival of intracranial PDX mice after recurring treatment with the indicated drugs. The x-axis represents survival from tumor implantation until moribund. For GBM39, remaining WSD-0922 treated mice were euthanized at the 100 day mark. (B) Representative MALDI-MS images showing drug measured in the intracranial tumor and surrounding regions. Images are accompanied by corresponding H&E staining. (C) Quantification of measured drug levels in the tumor core and rim, and surrounding normal tissue of the MALDI-MSI images in (B). (D) The concentration of drug in the plasma of each mouse was measured as described in 2C. (E) Brain-to-plasma and unbound brain-to-plasma concentration ratios were determined for GBM12 intracranial mice using the plasma levels in (D) and drug concentrations measured in the normal brain.

We performed a long-term survival study using mice implanted with intracranial tumors derived from the aforementioned patient lines. We utilized mice treated with erlotinib as a point of comparison since erlotinib has modest efficacy for GBM survival in preclinical studies, and failed to improve GBM patient survival in clinical trials.^24^ WSD-0922 provided a modest improvement in survival for GBM6 and GBM12 mice compared to vehicle and erlotinib-treated mice. However, GBM39 mice survived for almost twice as long with WSD-0922 (**Figure 3A**), with 60% of the mice surviving until the end of the study (day 100). Importantly, all of the adverse events recorded for GBM39 were due to toxicity associated with the drug and not tumor growth. We initially speculated that this striking difference in GBM39 survival might be due to improvement in drug delivery or BBB penetrance.

Drug distribution to tumor as determined by MALDI-MSI did not support this hypothesis, with similar drug concentrations of erlotinib and WSD-0922 measured in the tumor core, rim, and in normal brain tissues (**Figure 3B-C,****Supplementary Figure 6 and Table 2**). Moreover, the concentration of drug measured in GBM39 mice was not appreciably more than the concentration present in GBM6 and GBM12, which lacked the same robust response. Pharmacology of the 2 inhibitors also failed to provide a clear answer for the longer survival of GBM39 mice. Consistent with our data from flank tumor models, we observed lower levels of WSD-0922 in the plasma of mice with intracranial tumors compared to erlotinib (**Figure 3D**, **Supplementary Table 3**), but the brain-to-plasma concentration ratio (2 hours post-dose) measured using normal mouse tissue was significantly greater than that of erlotinib (**Figure 3E**). The utility of higher WSD-0922 BBB penetrance was tempered by a lower unbound concentration ratio (**Figure 3E**), suggesting that although more WSD-0922 is entering the brain, binding to surrounding brain tissue diminishes the concentration of free drug available to bind EGFR and other targets at the tumor site and in disseminated tumor cells. The net effect of these factors is similar concentrations of total WSD-0922 and erlotinib being measured in the tumor region. Our findings indicate that drug distribution into brain and tumor alone cannot explain the observed differences in survival, and led us to probe the tumor signaling response to WSD-0922 and erlotinib for additional insight.

### Phosphoproteomics Reveals a Differential Adaptive Signaling Response to WSD-0922

**Figure 4.**
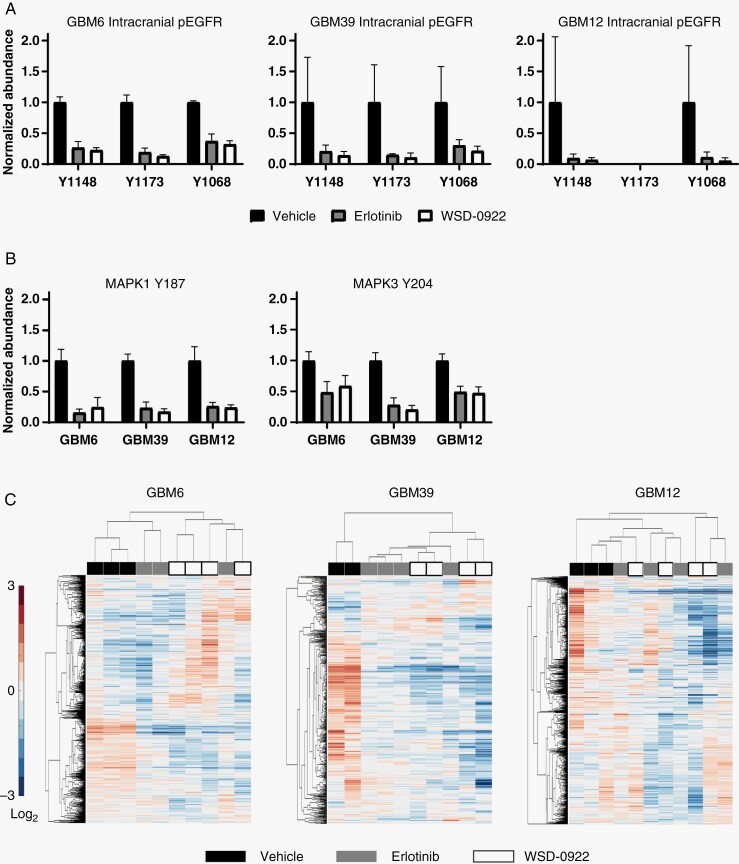
Evaluating the effect of WSD-0922 on intracranial epidermal growth factor receptor (EGFR) signaling. (A) Phosphoproteomics was used to measure the inhibition of EGFR peptide phosphorylation in intracranial tumors, as in 2D. Tyr 1173 pEGFR data were not included for GBM12 because TMT reporter ions for this phosphorylation event were below the mass spectrometer limit of detection in runs performed for these samples. (B) pERK1/2 levels were measured as in (A). (C) Heatmaps showing the hierarchical clustering of tumor samples (columns) and phosphopeptides (rows). Heatmaps reflect changes in the relative abundance of tyrosine-phosphorylated peptides in response to the indicated treatments. Values are shown as log_2_-fold changes over the mean abundance across all conditions for a given phosphopeptide.

To quantify the signaling network response to these EGFR inhibitors, we processed tumors from the intracranial mouse models and performed phosphotyrosine enrichment mass spectrometry. Similar to our observation in flank tumor models, WSD-0922 and erlotinib both significantly inhibited EGFR phosphorylation at several sites and ERK1/2 phosphorylation (**Figure 4A-B****Supplementary Figure 3**). The inhibition of the EGFR signaling network was further evident from distinct visible clusters of downregulated phosphopeptides in hierarchically clustered heatmaps (**Figure 4C**), including multiple phosphorylation sites in the canonical EGFR signaling network (**Supplementary Table 4**). Importantly, pEGFR inhibition was similar in each PDX model, suggesting that prolonged survival for GBM39 was not solely related to the EGFR signaling axis.

**Figure 5.**
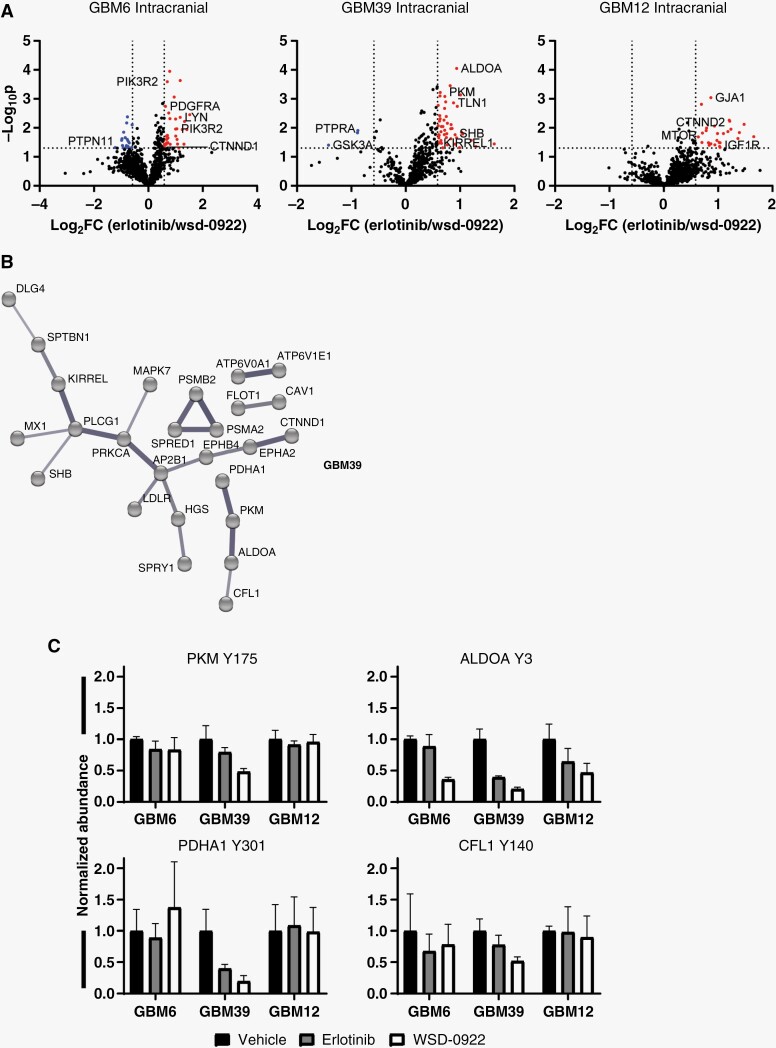
The broader target specificity of WSD-0922 enhances efficacy in GBM39. (A) Volcano plots were generated to show the differential adaptive response of intracranial patient derived xenograft tumors to WSD-0922 and erlotinib. Data points represent the log_2_ fold change (FC) of the average abundance in response to erlotinib over the WSD-0922 average abundance of a given phosphopeptide ($\log_{2}(erlotinib/WSD-0922)$). Dotted lines represent cutoffs for meaningful differences in abundance: log_2_FC with an absolute value ≥ 0.585, and –log_10_*P*-value ≥ 1.3. (B) STRING diagram of GBM39 phosphopeptides that had differential abundance in response to erlotinib and WSD-0922. Interactions with medium confidence from all interaction sources except “Textmining” are shown, and disconnected nodes were hidden for clarity. (C) Plots showing the abundances of select phosphopeptides from a subnetwork in B.

We then focused our attention on phosphoproteins that were differentially affected by WSD-0922 and erlotinib. We postulated that the survival difference observed in GBM39 might be driven by WSD-0922 providing better inhibition of certain kinases compared to erlotinib. Across the different PDX lines, we observed decreased phosphorylation of several proteins in WSD-0922 treated tumors compared to those treated with erlotinib (**Figure 5A**, **Supplementary Table 4**). We further captured the relationship between phosphoproteins that were differentially affected by erlotinib and WSD-0922 using STRING networks (**Figure 5B**, **Supplementary Figure 5B**). GBM6 tumors demonstrated a more traditional inhibitor-perturbed signaling network, with WSD-0922 more effectively inhibiting PDGFRα signals converging in downstream effectors such as Lyn and p85β. In contrast, GBM12 had a much more diffuse network, with IGF-1R phosphorylation as the most noteworthy protein whose phosphorylation was more inhibited by WSD-0922. Strikingly, the STRING diagram for GBM39 tumors contained a subnetwork of metabolic enzymes and the SFK substrate cofilin-1. Phosphorylation events on PKM (Tyr 175), PDHA1 (Tyr 301), ALDOA (Tyr 3), and cofilin-1 (Tyr 140) were decreased more with WSD-0922 than erlotinib, and importantly these changes were largely constrained to GBM39 and did not occur in the other PDX models (**Figure 5C**). Most of these phosphorylation sites have been previously shown to be regulated by EGFR,^25,26^ and SFK regulation of metabolic enzyme phosphorylation is well established.^27,28^ Given this interplay, we hypothesized that combination treatment with the SFK inhibitor dasatinib would augment the effectiveness of erlotinib in GBM39 mice. However, in a long-term survival study, single-agent erlotinib mice survived as long as mice treated with the combination (**Supplementary Figure 5C**). This result suggests that additional targets of WSD-0922 may be critical in determining survival, along with its effect on SFK and metabolic enzymes.

## Discussion

Despite a breadth of preclinical efforts and successful use for other cancer types, EGFR inhibition has not translated into an effective clinical approach for GBM. The likely reasons for these clinical failures are manifold. The BBB is perhaps the biggest obstacle to successful delivery of drugs to the tumor site. Most current anti-EGFR therapies have poor BBB-penetrance, and thus their limited effectiveness may be due in part to insufficient drug delivery.^6,29^ Previous studies have shown that the BBB can prevent drug levels in the brain from reaching the requisite concentration for tumor killing.^30^ The present study sought to overcome this challenge by using a more BBB-penetrant EGFR inhibitor. We consistently measured WSD-0922 total concentrations greater than 10 μM in the tumor core region, well above the ex vivo EC50 of ~200 nM (**Figure 1C**). We confirmed WSD-0922 is more penetrant than erlotinib in GBM12 mouse brains (**Figure 3**). However, the concentration of free WSD-0922 was significantly less than the concentration of free erlotinib (**Figure 3E**). The brain-to-plasma concentration ratio of WSD-0922 (0.356 at 2 hours post-dose) was comparable to that of gefitinib (0.358), which was speculated to have some BBB permeability.^16,31^ Yet, the WSD-0922 brain-to-plasma ratio is less than the values for strongly CNS-penetrant EGFR inhibitors like zorifertinib and osimertinib (1.7 and 0.988, respectively), and the unbound brain-to-plasma concentration ratio for WSD-0922 was lower than that of several other EGFR inhibitors.^16^ Our data underscore the importance of comprehensively evaluating the PK/PD for novel compounds, and suggest additional studies are warranted to better understand WSD-0922 nonspecific binding at the site of action.

WSD-0922 effectively inhibited EGFR signaling in all three GBM PDX models. We observed comparable inhibition of the EGFR signaling network by WSD-0922 and erlotinib in flank tumor models, where BBB considerations are not pertinent (**Figure 2D**, **Supplementary Figure 3**), and in intracranial tumors (**Figure 4**). Previous work has suggested that earlier generations of EGFR inhibitors had poor affinity for the EGFR mutations found in GBM, such as the vIII truncation.^7^ Our data does not support this claim, with both erlotinib and WSD-0922 effectively ablating WT and mutant EGFR phosphorylation.

Our results point to additional signaling adaptations that are common hallmarks of EGFR therapeutic resistance. In response to EGFR inhibition, cancer cells often rely on other RTKs for bypass signaling.^32,33^ Consistent with this finding, intracranial tumors showed increased phosphorylation of IGF-1R and PDGFRα after WSD-0922 and erlotinib treatment (**Supplementary Table 4**). Encouragingly, the phosphorylation of several sites on these proteins was lower with WSD-0922 treatment compared to erlotinib, providing additional evidence that the affinity of WSD-0922 for other protein targets enhances its efficacy against the cancer cell adaptive response.

A critical question raised by this study concerns the factors underlying the apparent differential drug sensitivity of the three PDX models. Aside from the distinct signaling networks, there are additional considerations that may explain the greater survival benefit in GBM39. For instance, based on previous work from our group, GBM12 has 100 times fewer copies of EGFR than GBM6 and GBM39,^17^ and thus may be inherently less responsive to anti-EGFR therapy. Additionally, there were a few EGFR phosphorylation sites that were minimally responsive to drug treatment in GBM6 flank and intracranial models: Tyr 954, Tyr 974, and Tyr 992 (**Supplementary Figure 3**). Each of these sites is within the C-terminal tail region of EGFR, and they are phosphorylated upon EGFR stimulation or activation.^34^ The incomplete de-phosphorylation of these sites suggests some residual EGFR activity in GBM6 that may signal through known adaptors such as PLCγ, and contribute to partial resistance.^35^

A distinguishing feature of the GBM39 signaling response to EGFR inhibition was the decreased phosphorylation of sites on metabolic enzymes (**Figure 5C**). Proteins such as PKM and PDHA1 have been shown to play a prominent role in influencing cancer cell metabolism.^36,37^ Our data suggest that these enzymes are basally phosphorylated at a higher level in GBM39 than in GBM6 and GBM12, and may point to a broader metabolic signature predictive of response to EGFR TKIs. In support of this view, recent work has demonstrated the potential for GBM tumor classification based on metabolic attributes.^38^ The exact mechanism underlying the link between cancer cell metabolism and susceptibility to EGFR inhibition remains unclear. A direct relationship was previously established between levels of EGFR vIII and phosphorylation of PKM^25^ and intriguingly, EGF stimulation of U87 MG cells overexpressing WT or vIII EGFR resulted in Tyr 301 phosphorylation of PDHA1 and phosphorylation of the Src activation loop.^26^ Additional metabolic enzyme phosphorylation events have been shown to be sensitive to dasatinib treatment, providing further support for an EGFR-SFK-metabolism signaling axis.^39^ Consistent with this hypothesis, in addition to decreased phosphorylation of metabolic enzymes, we also observed decreased phosphorylation of the SFK substrate cofilin in response to WSD-0922 treatment. Efforts are currently underway to comprehensively evaluate the metabolic state of various GBM PDX tumor models, and to validate WSD-0922 targets within these tumors.

There have been very few therapeutic advances in the treatment of GBM over the past two decades. The standard of care remains tumor resection plus a combination of radiation and chemotherapy, and patient survival has remained largely unchanged.^40^ Although collective research efforts have yielded a comprehensive understanding of the genetics and drivers of the disease, precision medicine, and targeted approaches have had limited impact on GBM therapy. WSD-0922 demonstrated strong anti-EGFR efficacy in our preclinical models, and provided a substantial improvement in survival for GBM39. While there are outstanding questions concerning the PK/PD and mechanism of action of the drug, the potential to benefit even a subset of GBM patients supports ongoing clinical evaluation of WSD-0922.

## Supplementary Material

vdad066_suppl_Supplementary_Figure_S1

vdad066_suppl_Supplementary_Figure_S2

vdad066_suppl_Supplementary_Figure_S3

vdad066_suppl_Supplementary_Figure_S4

vdad066_suppl_Supplementary_Figure_S5

vdad066_suppl_Supplementary_Figure_S6

vdad066_suppl_Supplementary_Material

vdad066_suppl_Supplementary_Table_S1

vdad066_suppl_Supplementary_Table_S2

vdad066_suppl_Supplementary_Table_S3

vdad066_suppl_Supplementary_Table_S4
