# Supplementary figures and images for: WSD-0922, a novel brain-penetrant inhibitor of epidermal growth factor receptor, promotes survival in glioblastoma mouse models
Source: Neurooncol Adv. 2023 May 27;5(1):vdad066. doi: 10.1093/noajnl/vdad066 (PMC10263119; doi:10.1093/noajnl/vdad066)

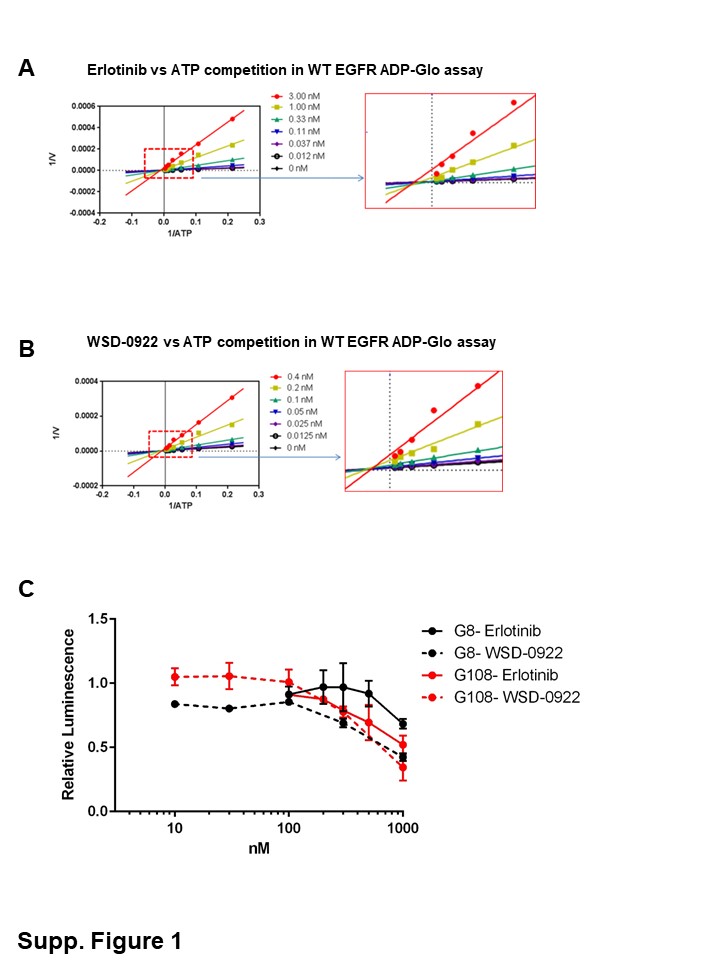

Supplement: vdad066_suppl_Supplementary_Figure_S1 [file vdad066_suppl_supplementary_figure_s1.jpeg]

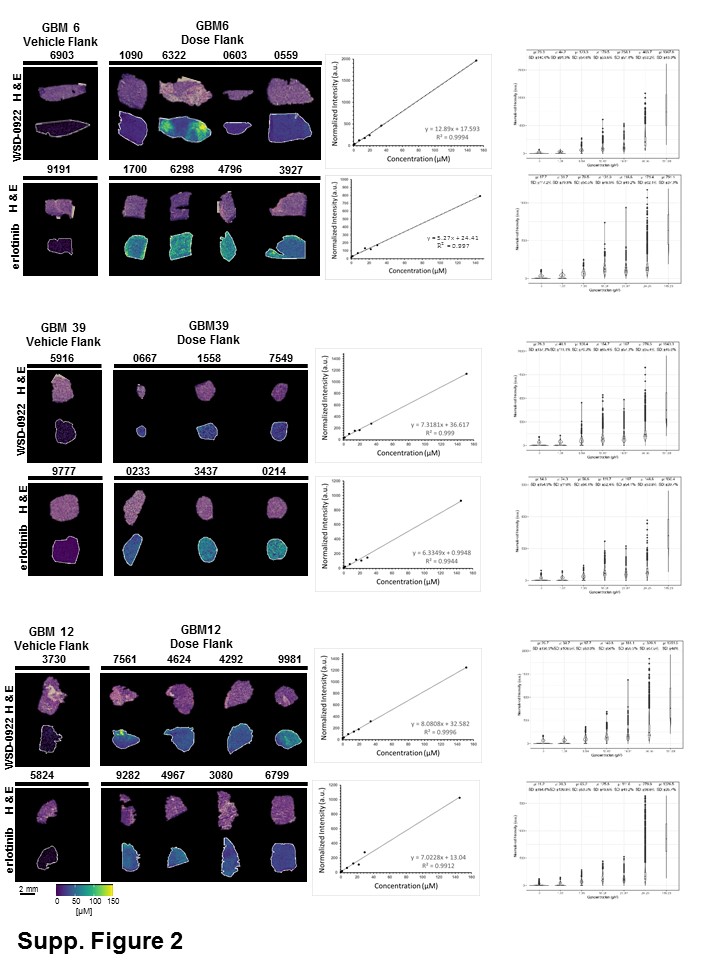

Supplement: vdad066_suppl_Supplementary_Figure_S2 [file vdad066_suppl_supplementary_figure_s2.jpeg]

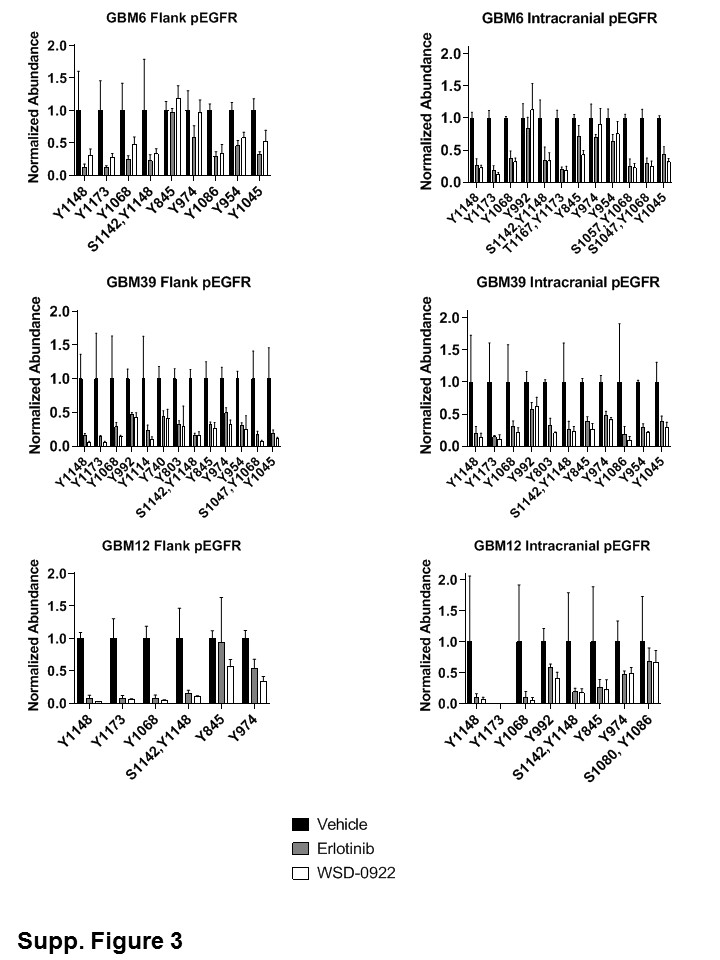

Supplement: vdad066_suppl_Supplementary_Figure_S3 [file vdad066_suppl_supplementary_figure_s3.jpeg]

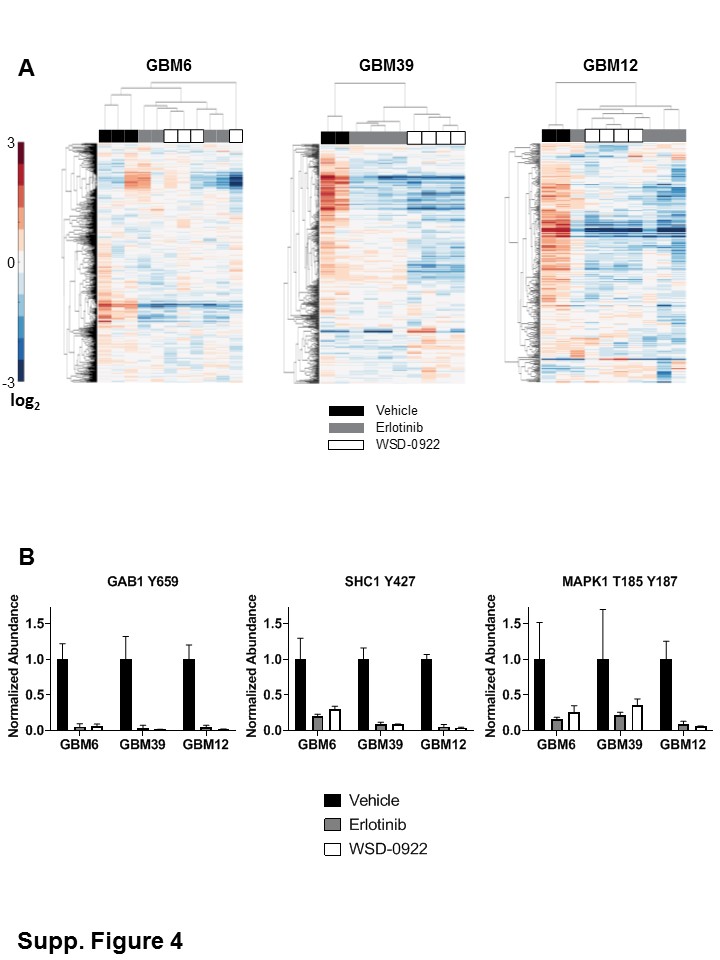

Supplement: vdad066_suppl_Supplementary_Figure_S4 [file vdad066_suppl_supplementary_figure_s4.jpeg]

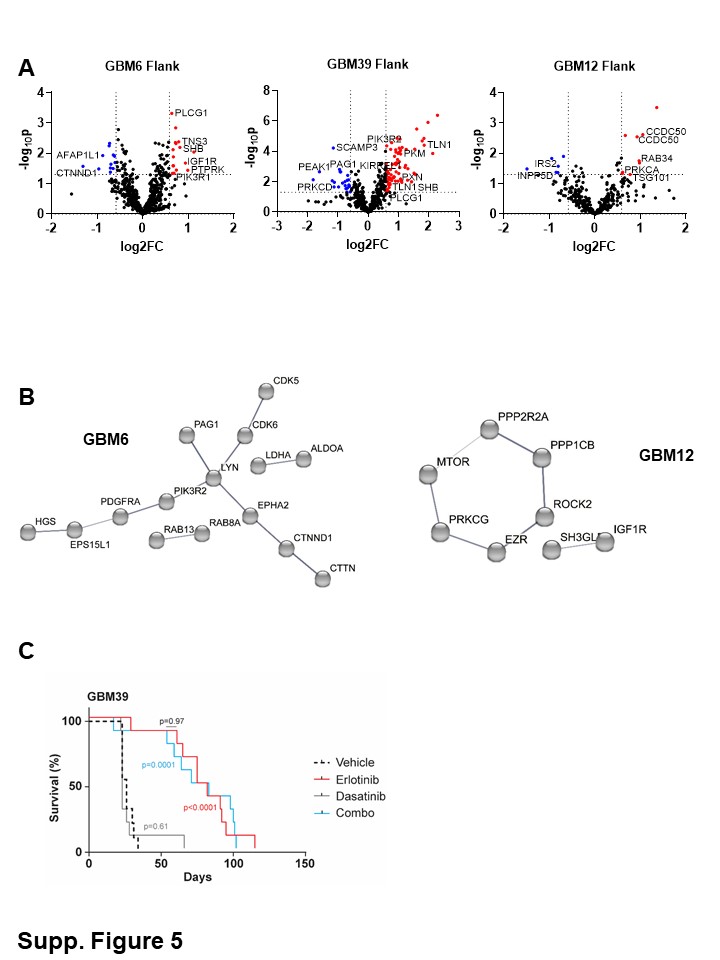

Supplement: vdad066_suppl_Supplementary_Figure_S5 [file vdad066_suppl_supplementary_figure_s5.jpeg]

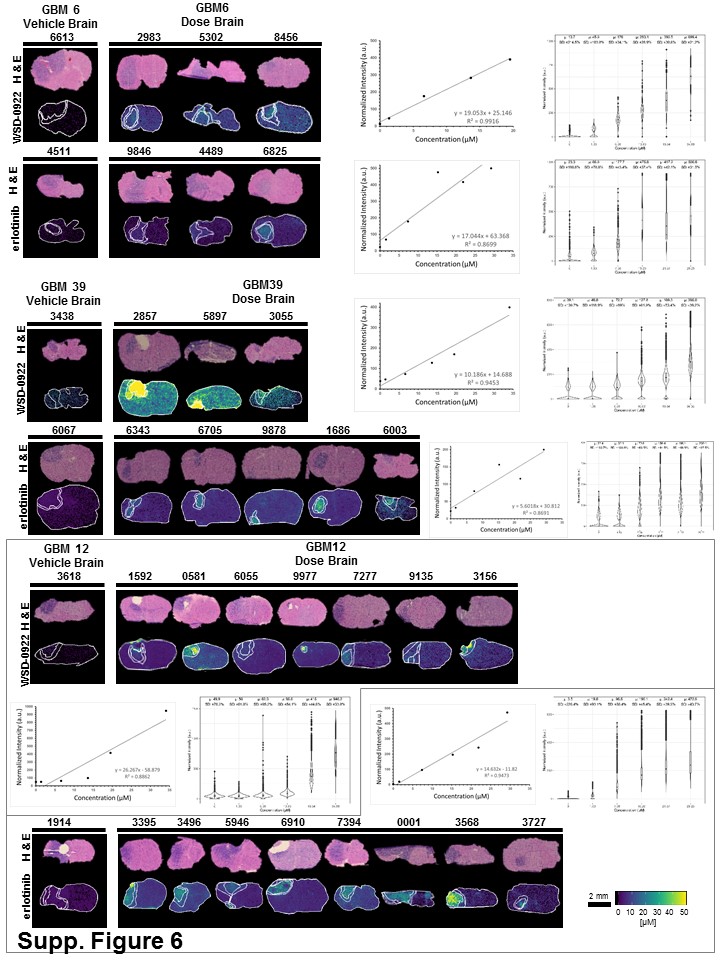

Supplement: vdad066_suppl_Supplementary_Figure_S6 [file vdad066_suppl_supplementary_figure_s6.jpeg]
