# Supplementary material for: WSD-0922, a novel brain-penetrant inhibitor of epidermal growth factor receptor, promotes survival in glioblastoma mouse models
Source: Neurooncol Adv. 2023 May 27;5(1):vdad066. doi: 10.1093/noajnl/vdad066 (PMC10263119; doi:10.1093/noajnl/vdad066)
Supplement: vdad066_suppl_Supplementary_Material [file vdad066_suppl_supplementary_material.docx]

**MATERIALS AND METHODS**

**Reagents**

WSD-0922 was provided by Wayshine Biopharm and stored at 4°C under desiccant conditions. Erlotinib was obtained from the Developmental Therapeutics Program at the National Institutes of Health.

**Cell Culture**

U87, U87 expressing wildtype EGFR (U87 EGFR WT) or the EGFR VIII deletion mutant (U87 EGFR VIII) were a kind gift from Dr. Frank Furnari (Department of Pathology, University of California- San Diego). Cells were grown in high glucose-DMEM (Corning 15013CV) containing 10% FBS (Atlanta Biologicals S11150) and 1% Penicillin/Streptomycin (Corning 30001CI) in a 5% CO2, humidified incubator at 37°C.

Explant cultures derived from Mayo Glioblastoma (GBM) patient-derived xenograft (PDX) flank tumors were cultured under serum-free conditions (StemPro Neural Stem Cell Serum Free Medium, Thermo Fisher Scientific A10509-01, supplemented with 4mM L-Glutamine (Corning 25-005-CI) and 1% Penicillin/Streptomycin (Corning 30001CI) on laminin (Sigma L2020-1mg) -coated tissue culture flasks.

The U87 cell line was authenticated using Short Tandem Repeat (STR) analysis from GeneCopoeia in 2021. STR is also performed on cells from each *in vivo* study, and periodically on the established PDX lines.

**ADP-Glo Assays**

The ADP-Glo Assay Kit (Promega V9102) was used to measure the enzymatic activity of EGFR and assess the IC_50_ and binding mode of erlotinib and WSD-0922 through monitoring of ATP conversion to ADP. Briefly, 1.5-10 nM of recombinant WT (Invitrogen PV3872) or mutant (Invitrogen PV6178/PV4128) EGFR was incubated with assay buffer, drug, and Poly(Glu, Tyr) substrate (Sigma P0275) in a 384 well plate. Upon ATP addition, the assay was performed per the manufacturer’s instructions, and luminescent measurements were recorded using Envision (Perkin Elmer).

**Kinase Panel Screening of WSD-0922**

The inhibition potency of WSD-0922 was tested against 205 kinases. Briefly, kinase reactions were performed in a 384 well plate with recombinant versions of each kinase and 1.5 μM of an appropriate fluorescently labeled FLPeptide substrate (Perkin Elmer). Wells received DMSO or 1 μM of WSD-0922 or the reference compound staurosporine. Plates were read for the conversion ratio using the EZ Reader system (Perkin Elmer), and %inhibition was determined.

**Western Blotting**

1.5e6 cells were plated in 10 cm tissue culture (TC) dishes and incubated overnight before a 24 h treatment with a concentration range of erlotinib (NCI) or WSD-0922. Cells were lysed in a buffer containing 50 mM Tris HCl, pH 7.4, 150 mM NaCl, 1% NP-40, 0.5% Na Deoxycholate, 1 mM EGTA, and 1 mM sodium fluoride, and incubated for 10 min on ice before centrifugation to remove debris. The supernatant was stabilized with LDS Sample Buffer (Invitrogen NP0007). After adding the reducing agent and heating for 10 min at 70°C, 15 μg of lysate was loaded into each well of a 4-12% gradient SDS-PAGE gel (Invitrogen NP0329BOX) and electrophoresed at 100 V for approximately 1.5 – 2 h. Gels were wet transferred to a PVDF membrane (Millipore Sigma IPVH00010) at 150 V for 1 h before membranes were blocked in 5% non-fat milk in Tris-buffered saline containing 0.1% Tween-20 (TBS-T) for 30 min at room temperature (RT). Membranes were incubated with 1:1000 dilutions of primary antibodies in 5% bovine serum albumin (BSA) in TBS-T overnight at 4°C on a rotating platform shaker before being washed at RT three times for 5 min each with TBS-T. Membranes were then incubated with an HRP-conjugated secondary antibody diluted at 1:5000 in 5% non-fat milk in TBS-T for 1 h at RT before three TBS-T washes as above. Target proteins were detected using an enhanced chemiluminescent (ECL) reagent (Thermo Fisher Scientific 34580) and imaged on an Azure Biosystems c600 imager. Antibodies were obtained from Cell Signaling Technologies: p-EGFR (Y1173) #4407; pEGFR (Y1068) #2236; Vinculin #13901; Anti-rabbit IgG, HRP-conjugated #7074; Anti-mouse IgG, HRP-conjugated #7076, or Abcam: (EGFR #ab32562).

**Cell TiterGlo Viability Assay**

500 (GBM12) or 2000 (GBM6, GBM39) cells were plated per well in a black-walled, 96-well plate (Corning) and incubated overnight under standard tissue culture conditions as stated above, before a 14-day treatment with erlotinib or WSD-0922. *In vitro* efficacy was measured using the Cell-TiterGLO 3D Viability Assay (Promega G9682) according to the manufacturer’s instructions and a Tecan Infinite M200 Pro plate reader capable of reading luminescence.

**Phosphotyrosine Data Analysis**

Raw mass spectral data files were processed as previously described^1^ with the following changes: 1) Raw files were processed with Proteome Discoverer version 2.5.2 (ThermoFisher Scientific) and searched against the human SwissProt database using Mascot version 2.4 (Matrix Science); 2) Peptide spectrum matches (PSMs) for phosphopeptides were filtered for ion score ≥ 20, and precursor isolation interference (<35%), and PSMs for the most abundant peptides in IP supernatant runs were filtered for ion score ≥ 25. Data was transformed as previously described using IP supernatants run on the Q-Exactive Plus^1^. For each phosphopeptide, relative quantification was represented as a ratio between TMT ion intensities for each tumor and the appropriate normalization channel or control. The relative abundance for each phosphopeptide was log_2_-transformed and visualized using MATLAB (version R2021b, Bioinformatics Toolbox version 4.15.2, MathWorks). Data were plotted with the ‘clustergram’ function with hierarchical clustering using ‘correlation’ distance. Relative abundances for select phosphopeptides were plotted using GraphPad Prism (version 9.3.1). Phosphopeptides that were differentially induced by erlotinib or WSD-0922 were identified using the following criteria: log_2_(erlotinib abundance/WSD-0922 abundance) ≥ 0.585 or ≤ -0.585; a p-value ≤ 0.05 for two-tailed, two-sample unequal variance Student’s t-test. STRING network analysis was performed on differential peptides^2^. Interactions with medium confidence from all interaction sources except ‘Textmining’ are shown, and disconnected nodes were hidden for clarity. Networks were further prepared for publication using Adobe Illustrator.

Supplementary Table 4 has a detailed list of abundances and the amino acid sequence of tyrosine phosphopeptides from each mass spec run. The raw mass spectrometry data and associated tables have been deposited to the ProteomeXchange Consortium via the PRIDE partner repository with the dataset identifier PXD032974. Data can be accessed for manuscript review with the following credentials: Username: reviewer_pxd032974@ebi.ac.uk; Password: SMWHoejx .

**REFERENCES**

1. Marin BM, Porath KA, Jain S, et al. Heterogeneous delivery across the blood-brain barrier limits the efficacy of an EGFR-targeting antibody drug conjugate in glioblastoma. *Neuro Oncol*. 2021;23(12):2042-2053.

2. Szklarczyk D, Gable AL, Lyon D, et al. STRING v11: protein–protein association networks with increased coverage, supporting functional discovery in genome-wide experimental datasets. *Nucleic Acids Res*. 2019;47(D1):D607-D613.

**SUPPLEMENTARY FIGURES**

**Supplementary Figure 1. Enzyme kinetic data for the binding mode of WSD-0922.** Representative Lineweaver-Burk plots for erlotinib **(A)** and WSD-0922 **(B)**. Experiment was performed twice for each compound. **(C)** Cell TiterGlo was used to assess the impact of erlotinib and WSD-0922 on the viability of GBM8 and GBM108 in ex vivo culture. Assay was performed as described in Figure 1C.

**Supplementary Figure 2. Quantification of drug distribution in flank tumors.** MALDI-MS images showing distribution of erlotinib and WSD-0922 within additional flank tumors not shown in Figure 2B. MALDI-MS images are accompanied by corresponding H&E staining. Graphs represent linear regression models used to perform pixel-wise calibration and generate images with absolute concentration values at each pixel for comparison of quantitative distribution of the two drugs.

**Supplementary Figure 3. EGFR phosphorylation in GBM PDX tumors in response to erlotinib or WSD-0922 treatment.** Phosphoproteomics was used to measure the inhibition of EGFR signaling within flank and intracranial tumors. Graphs reflect all tyrosine-phosphorylated EGFR sites detected in mass spectrometry runs for the listed PDX models. Bars represent the relative abundance of phosphopeptides containing the indicated EGFR tyrosine phosphorylation sites upon drug treatment. Values are normalized to the average abundance for the vehicle treated tumors.

**Supplementary Figure 4. Phosphoproteomic analysis of erlotinib and WSD-0922 treated flank tumors. (A)** Heatmaps showing the hierarchical clustering of tumor samples (columns) and phosphopeptides (rows). Heatmaps reflect changes in the relative abundance of tyrosine-phosphorylated peptides in response to the indicated treatments. Values are shown as log_2_-fold changes over the mean abundance across all conditions for a given phosphopeptide. **(B)** Phosphoproteomics was used to measure the inhibition of downstream EGFR signaling, assessed by phosphorylation of GAB1, SHC1, and MAPK1 within flank tumors.

**Supplementary Figure 5. PDX tumors demonstrate a differential adaptive response to erlotinib and WSD-0922. (A)** Volcano plots were generated as in 5A to show the differential adaptive response of flank PDX tumors to WSD-0922 and erlotinib. **(B)** STRING diagram of GBM6 and GBM12 intracranial phosphopeptides that had differential abundance in response to erlotinib and WSD-0922. **(C)** Kaplan-Meier curve showing the long-term survival of intracranial PDX mice after recurring treatment with the indicated drugs.

**Supplementary Figure 6. Quantification of drug distribution in intracranial tumors.** MALDI-MS image analysis was performed as in Supplementary Figure 2 on additional intracranial tumors not shown in Figure 3B.
