# Supplementary material for: WSD-0922, a novel brain-penetrant inhibitor of epidermal growth factor receptor, promotes survival in glioblastoma mouse models
Source: Neurooncol Adv. 2023 May 27;5(1):vdad066. doi: 10.1093/noajnl/vdad066 (PMC10263119; doi:10.1093/noajnl/vdad066)
Supplement: vdad066_suppl_Supplementary_Table_S1 [file vdad066_suppl_supplementary_table_s1.docx]

Supplementary Table 1. WSD-0922 and erlotinib inhibition of WT EGFR

|  |  | **IC_50_ (nM)** | | | |
| --- | --- | --- | --- | --- | --- |
| **Enzyme** | **Drug** | **Replicate**  **1** | **Replicate**  **2** | **Replicate**  **3** | **Average ± SD** |
| WT  EGFR | erlotinib | 0.125 | 0.140 | 0.120 | 0.128 ± 0.011 |
|  | WSD-0922 | 0.058 | 0.051 | 0.058 | 0.056 ± 0.004 |
